# Decoding of Motor Coordination Imagery Involving the Lower Limbs by the EEG-Based Brain Network

**DOI:** 10.1155/2021/5565824

**Published:** 2021-06-23

**Authors:** Yunfa Fu, Zhouzhou Zhou, Anmin Gong, Qian Qian, Lei Su, Lei Zhao

**Affiliations:** ^1^Faculty of Information Engineering and Automation, Kunming University of Science and Technology, Kunming 650500, China; ^2^Brain Cognition and Brain-computer Intelligence Integration Group, Kunming University of Science and Technology, Kunming 650500, China; ^3^Brain Science and Visual Cognition Research Center, School of Medicine, Kunming University of Science and Technology, Kunming 650500, China; ^4^Yunnan Provincial Key Laboratory of Computer Technology Applications, Kunming, China; ^5^School of Information Engineering, Chinese People's Armed Police Force Engineering University, Xian 710000, China; ^6^Faculty of Science, Kunming University of Science and Technology, Kunming 650500, China

## Abstract

Compared with the efficacy of traditional physical therapy, a new therapy utilizing motor imagery can induce brain plasticity and allows partial recovery of motor ability in patients with hemiplegia after stroke. Here, we proposed an updated paradigm utilizing motor coordination imagery involving the lower limbs (normal gait imagery and hemiplegic gait imagery after stroke) and decoded such imagery via an electroencephalogram- (EEG-) based brain network. Thirty subjects were recruited to collect EEGs during motor coordination imagery involving the lower limbs. Time-domain analysis, power spectrum analysis, time-frequency analysis, brain network analysis, and statistical analysis were used to explore the neural mechanisms of motor coordination imagery involving the lower limbs. Then, EEG-based brain network features were extracted, and a support vector machine was used for decoding. The results showed that the two employed motor coordination imageries mainly activated sensorimotor areas; the frequency band power was mainly concentrated within theta and alpha bands, and brain functional connections mainly occurred in the right forehead. The combination of the network attributes of the EEG-based brain network and the spatial features of the adjacency matrix had good separability for the two kinds of gait imagery (*p* < 0.05), and the average classification accuracy of the combination feature was 92.96% ± 7.54%. Taken together, our findings suggest that brain network features can be used to identify normal gait imagery and hemiplegic gait imagery after stroke.

## 1. Introduction

Approximately 45% of poststroke hemiplegic patients depend on wheelchairs to move [1–4]. Unfortunately, traditional physical therapy has only a limited effect on rehabilitation of the lower limbs of poststroke hemiplegic patients [5]. In contrast, motor imagery therapy can induce plasticity in the brain [6–10], which can partially restore lower limb movement in poststroke hemiplegic patients [11–14]. At present, most motor imagery therapies have focused on improving the performance of the upper limbs [15, 16], whereas only a few studies have aimed at improving performance of the lower limbs [17]. In addition, current and past studies on motor coordination have mainly focused on control of redundant degrees of freedom in motor coordination [18–23], quantification of motor coordination [24–29], external environments [30], and evaluation methods of motor coordination [31–34]. However, few studies have investigated the neural mechanisms of motor coordination imagery, and there has not been much focus on decoding motor coordination imagery involving the lower limbs.

For the decoding of motor coordination imagery, conventional features have mainly adopted time-domain features [35], frequency-domain features [36], time-frequency features [37], spatial-domain features [38], and fusion of time-frequency-spatial multidomain features [39]. These traditional features mainly reflect the activation level of the corresponding brain area. However, motor coordination imagery of the lower limbs involves not only the activation of related brain regions but also the coordinated control among related brain regions [36]. Therefore, it is necessary to use brain network features to more comprehensively analyze the neural correlates of motor coordination imagery.

In the present study, we proposed a new paradigm (normal gait imagery and hemiplegic gait imagery after stroke) of motor coordination imagery involving the lower limbs. In our paradigm, normal gait imagery is abbreviated as motor coordination imagery (MCI), and poststroke hemiplegic gait imagery is abbreviated as imaging exceptional gait (IEG). In addition to traditional time-domain analysis, power spectrum analysis, and time-frequency analysis, a method for brain network analysis was also used to investigate the neural mechanisms and decoding of motor coordination imagery involving the lower limbs.

The rest of the paper is organized as follows. Details of the method are illuminated in Section 2 including experiments configuration and data analysis.  Section 3 displays the brain topographic maps, functional brain networks during MCI, IEG, and REST, and comparison results of the average classification accuracy of MCI and IEG. In Section 4, a brief discussion of the results is given. Finally, Section 5 concludes the paper.

## 2. Methods

### 2.1. Subjects

Previous studies have shown that brain activity patterns evoked by motor imagery and actual exercise are similar [33]. Hence, our present study used the Kinesthetic and Visual Imagery Questionnaire (KVIQ) [38–50] to recruit 30 healthy subjects (numbered s1–s30) with strong motor imagery abilities (questionnaire score ≥ 70 points) to serve as participants for data collection. All recruited subjects were male, right-handed, without psychological disease and were 25 ± 1 years old. This study was approved by the Medical Ethics Committee of Kunming University of Science and Technology. Each subject signed an informed written consent before the start of the experiment.

### 2.2. Experimental Paradigm

**Figure 1:**
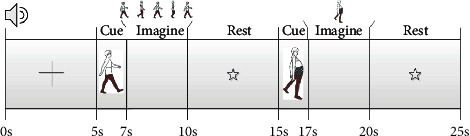
The timing of a single trial.

Coordinated movement is a type of exercise to restore and strengthen motor coordination. It usually involves the coordinated movement of multiple joints and limbs. Specifically, it includes motor coordination of the following: the upper and lower limbs, the limb trunk, and both symmetrical coordination and asymmetrical coordination of the left and right limbs. Among these components, coordinated movement of the upper limbs and hands can train the accuracies, reaction speeds, and rhythms of actions, while coordinated movement of the lower limbs can train correct gaits and motor coordination of the upper and lower limbs. In order to establish a method for recovering motor abilities of poststroke hemiplegic patients, the present study focused on motor coordination imagery of the lower limbs. We designed MCI and IEG. The timing of each trial is shown in Figure 1. The screen at the beginning of the experiment showed a fixed cross for 5 s, which prompted subjects to prepare for the experiment. When the fixed cross disappeared, the screen showed a prompt for normal gait imagery for 2 s, which prompted subjects to prepare for normal gait imagery. Once the prompt disappeared, the screen became blank and required the subjects to imagine a normal gait for 3 s. When the imagery period ended, the screen showed a star prompt for 5 s, indicating the subjects to rest. After the star prompt disappeared, the screen showed a prompt for poststroke hemiplegic gait imagery for 2 s, which prompted subjects to prepare for poststroke hemiplegic gait imagery. After the prompt disappeared, the screen became blank and required subjects to imagine a poststroke hemiplegic gait for 3 s. At the end of the imagery period, the screen showed a star prompt for 5 s, prompting the subjects to rest. This entire sequence comprised one experimental block, and each subject completed a total of 50 blocks.

### 2.3. Experimental Setup

**Figure 2:**
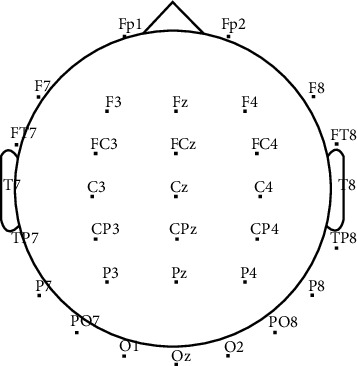
Schematic diagram of the electrode layout.

During the experiment, subjects were approximately 70 cm away from the prompt screen, and their hands were placed on the desktop. A TCL 24-inch LCD monitor was used for task prompts. A Lenovo ThinkPad computer running Windows 10 was used for data acquisition and processing via MATLAB software. The electroencephalogram (EEG) amplifier consisted of an NT9200 series (Beijing Zhongke Xintuo Instrument Co., Ltd.) with a sampling rate of 1000 Hz and 45 Hz low-pass filtering. At the beginning of the experiment, each subject completed the task according to the prompts in Figure 1. During the experiment, the subjects were required to avoid movements and blinking as much as possible during the visual imagery task.  Figure 2 shows the schematic diagram of the electrode layout. The collected EEG data consisted of the following channels: Fp1, Fp2, F7, F3, Fz, F4, F8, FT7, FC3, FCz, FC4, FT8, T7, C3, Cz, C4, T8, TP7, TP8, CP3, CP4, CPz, P7, P3, Pz, P4, P8, PO7, PO8, O1, O2, and Oz. The electrode layout included spatial positioning over the frontal lobe, parietal lobe, occipital lobe, and temporal lobe. The reference electrodes are denoted as A1 and A2.

### 2.4. Data Analysis

#### 2.4.1. Data Preparation

Visual inspection was carried out on the collected EEG data to remove any data segments with substantial noise in the EEG signal. Then, the EEG data of the 30 subjects under different task conditions were extracted.

#### 2.4.2. Pretreatment

First, baseline drift correction was performed on the extracted EEG signals to eliminate deviations of the EEG signal from the baseline [51]. An elliptic filter was used for 8–30 Hz band-pass filtering. Then, independent component analysis (ICA) was used to remove electrooculogram artifacts and electromyographical artifacts. Additionally, alpha, beta, gamma, zeta, delta, and theta bands were extracted.

#### 2.4.3. Time-Domain Analysis

After preprocessing, the data were divided into three categories: MCI, IEG, and process of rest (REST). Then, these three categories of data were stacked and averaged, and we compared the movement-related cortical potentials (MRCPs) among them in the time domain and compared the MRCP average across channels and the grand average across subjects among these three categories of data.

#### 2.4.4. Analysis of Brain Topography

In order to determine the activation location of the brain region under the three tasks of MCI, IEG, and REST, the three categories of data were preprocessed and then averaged, after which the brain topographies during the three tasks were determined at 1, 100, 200, 300, 400, and 500 ms.

#### 2.4.5. Power Spectrum Analysis

In order to analyze the EEG power associated with the task and reduce the impact of absolute power between subjects and electrodes, the absolute powers, ABSP, of MCI and IEG were obtained by using the power of MCI minus the power of REST, as well as by using the power of IEG minus the power of REST. The quantitative method for determining the power spectral density was via a fast Fourier transform algorithm. ABSP was calculated as follows [52]:

(1) $$\begin{array}{c} {\text{ABSP}}_{\text{MCI}}={\text{POWER}}_{\text{MCI}}-{\text{POWER}}_{\text{REST}},\\ {\text{ABSP}}_{\text{IEG}}={\text{POWER}}_{\text{IEG}}-{\text{POWER}}_{\text{REST}}. \end{array}$$

#### 2.4.6. Brain Network Analysis

In order to determine the connectivity of the brain function network under the three tasks of MCI, IEG, and REST, a multivariable autoregressive (MVAR) model was established to obtain a time-varying direct directed transfer function (dDTF). Because dDTF can eliminate the pseudocausal relationship derived from other leads [53], dDTF was used as a measure of causality, and Granger causality analysis was performed on the three types of preprocessed data to generate a dDTF weight matrix. The threshold value used in the present study was set to 0.2. Then, we generated an adjacency matrix under the selected threshold. Finally, we obtained the brain function network under the three types of tasks.

Specifically, the original EEG data, *X*(*t*), of the *N* lead can be expressed as follows:

(2) $$\begin{array}{c} X\left(t\right)={\left[X_{1}\left(t\right),X_{2}\left(t\right),\ldots ,X_{N}\left(t\right)\right]}^{T}. \end{array}$$

Among them, *X*_*i*_(*t*) is the EEG time series of the *i*th lead. MVAR can be constructed according to *X*(*t*) [54]:

(3) $$\begin{array}{c} X\left(t\right)=\overset{p}{\underset{k=1}{\sum }}A\left(k\right)X\left(t-k\right)+E\left(t\right). \end{array}$$

Among them, *A*(*k*) is a matrix of model coefficients of *N* × *N*, which represents the dependence of the time delay, *k*; *p* is the model order, and *E*(*t*) is the random noise. Our present study used the Bayesian information criterion to determine the model order, *p*.

By Fourier transform of *A*(*k*), we obtain

(4) $$\begin{array}{c} A\left(f\right)=-\overset{p}{\underset{k=0}{\sum }}A\left(k\right)e^{-i2\pi fk}. \end{array}$$

Furthermore, **X**(*f*) in the frequency domain can be obtained as follows:

(5) $$\begin{array}{c} X\left(f\right)=A{\left(f\right)}^{-1}E\left(f\right)=H\left(f\right)E\left(f\right). \end{array}$$

Among them, *H*(*f*) is the transfer matrix of the system.

Furthermore, the dDTF can be obtained as [55]

(6) $$\begin{array}{c} \chi_{ij}^{2}\left(f\right)=F_{ij}^{2}\left(f\right)C_{ij}^{2}\left(f\right). \end{array}$$

Among them, *F*_*ij*_^2^(*f*) is the full frequency DTF, and the calculation method is as follows:

(7) $$\begin{array}{c} F_{ij}^{2}\left(f\right)=\frac{{\left|H_{ij}\left(f\right)\right|}^{2}}{\sum_{f}\sum_{k=1}^{N}{\left|H_{ik}\left(f\right)\right|}^{2}}, \end{array}$$

where *C*_*ij*_(*f*) is the partial coherence. The calculation method is as follows:

(8) $$\begin{array}{c} C_{ij}\left(f\right)=\frac{M_{ij}\left(f\right)}{\sqrt{M_{ii}\left(f\right)M_{jj}\left(f\right)}}, \end{array}$$

where M_*ij*_ is a minor of spectral matrix (matrix of spectra and cross-spectra) with the *i*-th row and *j*-th column removed.

#### 2.4.7. Measurement Analysis of the Brain Function Network

In order to analyze the connectivity of the brain function network in detail, four kinds of network measures (characteristic path length, global efficiency, clustering coefficient, and local efficiency) were selected for comparative analysis of the three types of tasks. The characteristic path length, PL, and global efficiency, *E*_glob_, measure the global transmission capability of the network as follows [56]:

(9) $$\begin{array}{c} PL=\frac{1}{N\left(N-1\right)}\underset{i,j\in N,i\neq j}{\sum }d_{ij}, \end{array}$$

(10) $$\begin{array}{c} E_{\text{glob}}=\frac{1}{N\left(N-1\right)}\underset{i,j\in N,i\neq j}{\sum }d_{ij}. \end{array}$$

In equations (9) and (10), the distance between two nodes, *i* and *j*, in the network is represented by *d*_*ij*_, which represents the edge number information of the shortest path, and *N* is the total number of nodes in the network. It can be determined from formulas (9) and (10) that the calculation method of global efficiency is similar to the characteristic path length. The difference is that the global efficiency, *E*_glob_, does not need to consider the isolated points in the network, which is suitable for a regular network, small world network, or a random network.

The local efficiency *E*_loc_ measures the local transmission capacity of the network. The local efficiency *E*_loc_ of the network is the average of the global efficiency of the subgraphs corresponding to all nodes in the network:

(11) $$\begin{array}{c} E_{\text{loc}}=\frac{1}{N}\underset{i\in N}{\sum }E_{\text{glob}}\left(G_{i}\right). \end{array}$$

In equation (11), *G*_*i*_ represents the subnetwork formed by all nodes directly connected with node *i*.

The cluster coefficient *CL*_*a*_ measures the degree of grouping of the network. The cluster coefficient *CL*_*a*_ of a network is the mean value of the cluster coefficient *cl*_*i*_ of all nodes in the network, where the cluster coefficient of node *i* is defined as follows:

(12) $$\begin{array}{c} cl_{i}=\frac{2e_{i}}{k_{i}\left(k_{i}-1\right)}=\frac{\sum_{j,m}a_{ij}a_{im}a_{jm}}{k_{i}\left(k_{i}-1\right)}. \end{array}$$

In equation (12), ∑_*j*,*m*_*a*_*ij*_*a*_*im*_*a*_*jm*_ is the number of edges included in the subnetwork composed of node *i* and is directly connected to nodes *j* and *m*. *k*_*i*_(*k*_*i*_ − 1) is the maximum number of edges of the subnetwork, and *k*_*i*_ is the degree of node *i*. Therefore, the clustering coefficient *CL*_*a*_ of a network is as follows:

(13) $$\begin{array}{c} CL_{a}=\frac{1}{N}\overset{N}{\underset{i=1}{\sum }}cl_{i}. \end{array}$$

In equation (13), *N* represents the total number of nodes in the network.

### 2.5. Classification Verification

In order to verify the difference between normal motor coordination imagery involving the lower limbs and that of poststroke hemiplegia gait imagery, the time-domain MRCP feature, power spectrum feature, time-frequency feature, network attribute feature, combined features of network attributes, and the adjacency matrix space were extracted. Since the SVM classifier has a good classification effect on small sample data [57], SVM was used for classification verification.

Specifically, the MRCP feature was used to extract the peak and phase of the 32 electrodes and to form a 32-dimensional feature matrix. The feature of the power spectrum was used to for the AR model to estimate the power spectrum and to obtain a 32-dimensional feature matrix. The time-frequency feature was to extract the time-frequency energy of the 32-lead EEGs by HHT and to form a 32-dimensional feature matrix. The network attribute feature was used to calculate the characteristic path length of the 32 electrodes to form a 32-dimensional characteristic matrix. The spatial feature of the adjacency matrix was used to extract the 16-dimensional feature matrix of the adjacency matrix using a spatial filter.

### 2.6. Statistical Analysis

In order to test the significance of the task differences, the characteristic path lengths of 15 channels for the three types of tasks were compared in pairs by repeated-measures analysis of variance. The statistical significance level was set as *p* < 0.05.

## 3. Results

**Figure 3:**
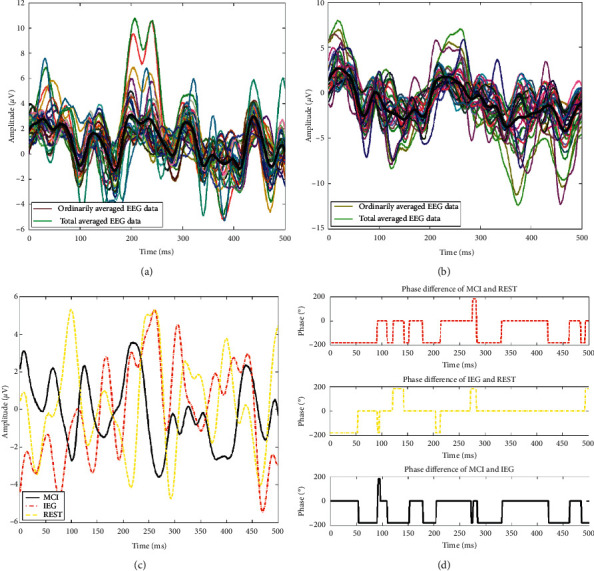
The average EEG waveforms across subjects, the average EEG across channels, and the phase difference of EEG data during MCI, IEG, and REST.

Figure 3(a) shows the average EEG waveforms across channels and the grand average across subjects during MCI. The colored line represents the average EEG data during MCI across all subjects, and the black line represents the grand average EEG data of MCI across the 32 channels. It can be seen from Figure 3(a) that a negative ERP was generated at about 200 ms. Figure 3(b) shows the average and grand average waveforms during IEG for all subjects. Figure 3(c) shows a comparison of the stack average of EEG data after preprocessing under MCI, IEG, and REST tasks. Figure 3(d) shows the phase difference of EEG data between MCI and REST, IEG and REST, and MCI and IEG. The EEG waveform trends of IEG and REST states were relatively consistent, while the EEG waveform trends of MCI and REST states were almost opposite to one another; additionally, the EEG waveform trends of MCI and IEG states were also opposite to one another (Figure 3(d)).

**Figure 4:**
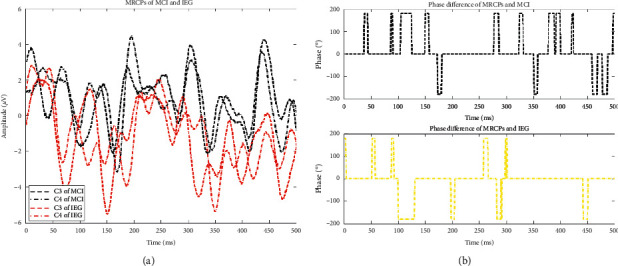
Differences in MRCPs and MRCP phases between MCI and IEG.

Figure 4(a) shows the MRCP difference between the MCI and IEG on the C3 and C4 channels, and Figure 4(b) shows the MRCP phase difference between the MCI and IEG on the C3 and C4 channels. Figure 4 shows that, for the MCI MRCPs, the waveforms of the C3 and C4 channels were similar, but there was a significant difference at about 200 ms. For the IEG MRCPs, the waveforms of the C3 and C4 channels were nearly opposite to one another, and the waveforms of the C3 channel were half a cycle ahead of those of the C4 channel, while they reached a consistency at about 200 ms.

**Figure 5:**
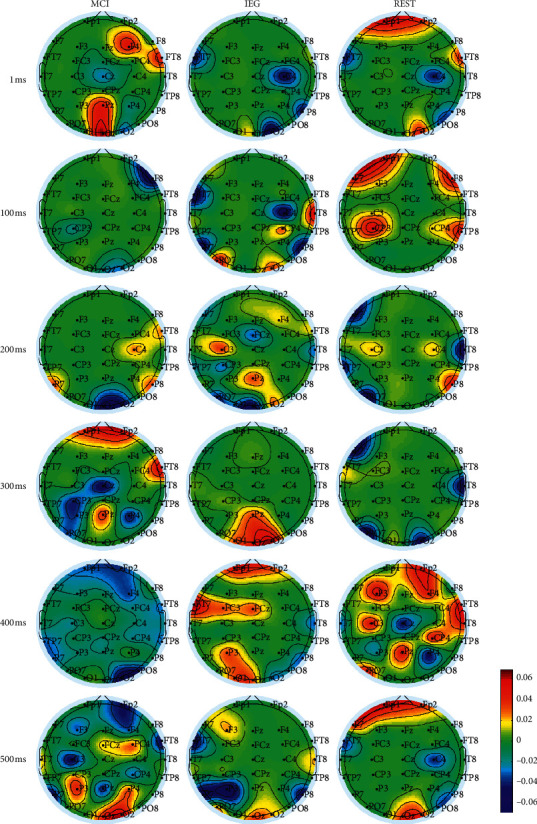
The brain topographic maps at 1, 100, 200, 300, 400, and 500 ms during MCI, IEG, and REST. The left, middle, and right columns show the brain topographic maps during MCI, IEG, and REST tasks, respectively. The brain topographic maps, from top to bottom, occurred at 1, 100, 200, 300, 400, and 500 ms during the corresponding task.

Figure 5 shows the brain topographic maps at 1, 100, 200, 300, 400, and 500 ms during MCI, IEG, and REST. At about 200 ms, during MCI, the brain topographic map was mainly activated in the right brain sensorimotor area (C4 channel); during IEG, the brain topographic map was mainly activated in the left brain sensorimotor area (C3 channel) and the parietal lobe (Pz channel), while during REST the brain topographic map was mainly activated in the left and right sensorimotor areas (C3 and C4).

In terms of timing, MCI first activated the right frontal lobe (F4 channel), left sensorimotor area (C3 channel), and parietal lobe (Pz channel) at 100 ms; the right sensorimotor area (C4 channel) was activated at 200 ms, whereas the left sensorimotor area (C3 channel) and parietal lobe (Pz channel) were activated at 300 ms. The central frontal area (FCz channel), central sensorimotor area (Cz channel), and right parietal lobe (P4 channel) were activated at 400 ms; the central areas of right frontal lobe (FC4 channel) and left parietal lobe (P3 channel) were activated at 500 ms. IEG first activated the left and right parietal central areas (CP3 and CP4 channels) at 100 ms; the left sensorimotor area (C3 channel) and the parietal lobe (Pz channel) were activated at 200 ms; the frontal central area (FCz channel) was activated at 300 ms. The left frontal central areas (FC3 channel), middle frontal lobe (FCz channel), and left parietal lobes (P3 channel) were activated at 400 ms; the left frontal lobe (F3 channel) was activated at 500 ms. REST first activated the central area of the left and right parietal lobes (CP3 and CP4 channels) at 100 ms; the left and right sensorimotor regions (C3 and C4) were activated at 200 ms. The left frontal lobes (F3 channel), left sensorimotor region (C3 channel), and the central area of the parietal lobes (Pz channel) were activated at 400 ms.

**Figure 6:**
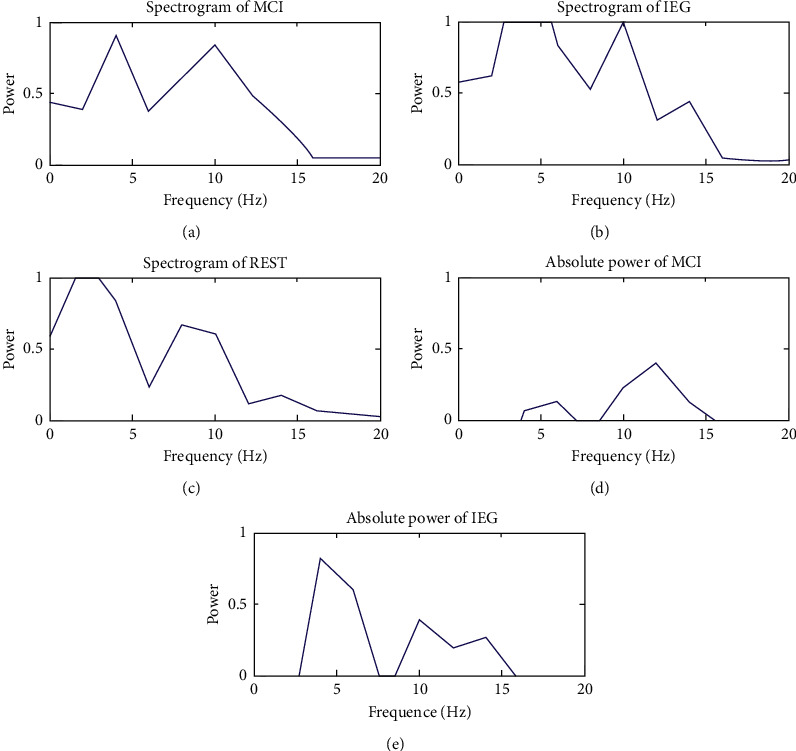
Comparison of absolute power changes during MCI and IEG.

Figure 6 shows the power spectrum during MCI, IEG, and REST tasks, as well as the absolute power spectrum of MCI and IEG. According to changes in the absolute power spectrum of MCI and IEG in Figures 6(d) and 6(e), the power distributions of MCI and IEG were in the *θ* wave (4–7 Hz), *α* wave (8–13 Hz), and *β* wave (14–16 Hz). The frequency band corresponding to the absolute power showed that the power related to MCI was mainly concentrated in the *α* wave, while the power related to IEG was mainly concentrated in the *θ* wave.

**Figure 7:**
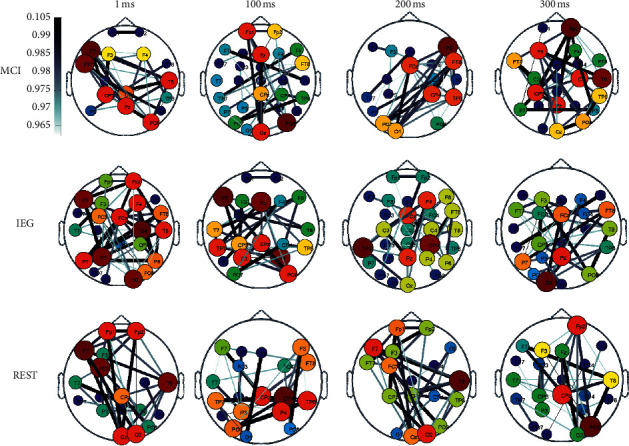
Functional brain networks at 1, 100, 200, and 300 ms during MCI, IEG, and REST. The upper, middle, and lower rows show the brain function networks during MCI, IEG, and REST, respectively. From left to right, the brain function networks occur at 1, 100, 200, and 300 ms during the corresponding task, respectively.

Figure 7 shows the brain function networks of MCI, IEG, and REST at 1, 100, 200, and 300 ms. The main core nodes of the brain network were F7, FT7, T8, CP3, Pz, and PO8 at 1 ms for MCI, and the network connection took place from the left frontal lobe to the right occipital lobe (Figure 7). The main core nodes of the brain network were FP1, Fz, Oz, and PO8 at 100 ms, and the network connection took place from the forehead to the occipital lobe. The main core nodes of the brain network were F8, FT8, FCz, CP4, TP8, PO7, and O1 at 200 ms. The main core nodes of the brain network were FP2, F3, FT7, T8, TP8, PO8, and Oz at 300 ms, and the network connection occurred from the right forehead to the right occipital lobe. At 1 ms, the main core nodes during IEG were F7, FP2, F4, FCz, C4, P3, and O2, and the network connection took place in the left and right hemispheres; at 100 ms, the main core nodes of the brain network were F7, Fz, P4, and PO8, and the network connection took place from the left forehead to the right occipital lobe. At 200 ms, the main core nodes of the brain network were F4, FCZ, CP4, TP7, and Pz, and the network connection took place in the right hemisphere; at 300 ms, the main core nodes of the brain network were FCz, FT8, P7, Pz, and O1, and the network connection occurred from right forehead to left occipital lobe. When REST was 1 ms, the main core nodes of brain network were FP1, FP2, F7, FC3, T8, O1, and O2, and the network connection took place in the left prefrontal lobe and the right occipital lobe; when REST was 100 ms, the main core nodes of the brain network were PO7, CPz, CP4, P4, and TP8, and the network connection took place in the left and right occipital lobes. At 200 ms during REST, the main core nodes of the brain network were F7, T8, and O2, and the network connection took place in the left prefrontal lobe; when REST was 300 ms, the main core nodes of the brain network were FP2, CPZ, and PO8, and the network connection occurred from the right forehead to the occipital lobe.

**Figure 8:**
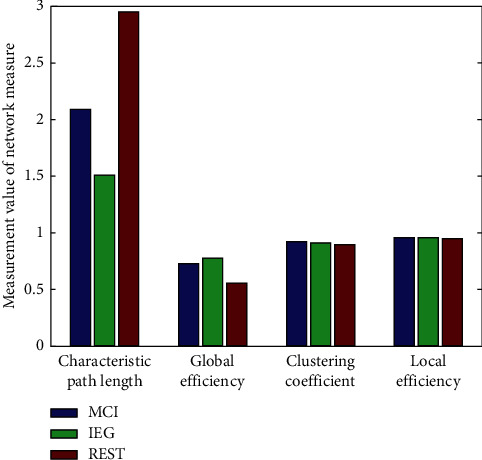
Comparison of network measures during MCI, IEG, and REST.

Figure 8 shows the comparison of brain network measures among MCI, IEG, and REST tasks. The four selected brain network measures were as follows: characteristic path length, global efficiency, clustering coefficient, and local efficiency. Among these four measures, the difference in the characteristic path lengths was the largest across MCI, IEG, and REST, while the other three measures exhibited only small differences across groups (Figure 8). REST had the largest characteristic path length, followed by MCI and finally IEG. The characteristic path length showed that the speed of information transmission between nodes in IEG was the fastest, followed by MCI and REST.

In order to statistically analyze differences among MCI, IEG, and REST, the characteristic path lengths of the 15 channels during MCI, IEG, and REST were selected for repeated-measures single-factor analysis of variance.  Table 1 shows the statistical results, where significant differences are marked in red. The difference in the characteristic path length between MCI and REST was significant, and only the O2 channel of MCI was not significantly different between MCI and REST (Table 1). There was no significant difference between IEG and REST. Only the *p* values of P3, C3, CP4, and Pz channel were small during IEG, and there was a significant difference between MCI and IEG.

**Figure 9:**
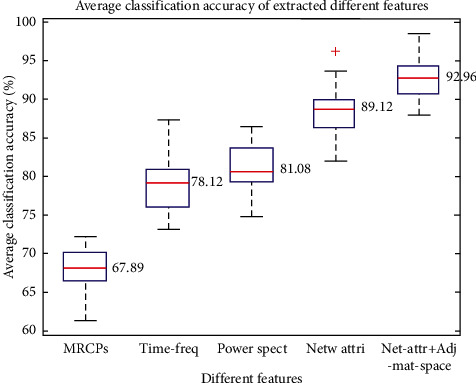
Comparison of the average classification results of MCI and IEG classified by MRCPs, time-frequency analysis, power spectrum analysis, network attribute analysis, a combination of the network attribute features, and the spatial features of the adjacency matrix.

Figure 9 shows the average classification results obtained by classifying MCI and IEG. The classifier adopted features of MRCPs, time-frequency analysis (Time-freq), power spectrum analysis (Power spec), network attribute analysis (Net-attr), a combination of the network attribute features, and the spatial features of the adjacency matrix (Net-attr + Adj-mat-space). The average classification accuracies of MRCPs, time-frequency analysis, power spectrum analysis, network attribute analysis, a combination of the network attribute features, and the spatial features of the adjacency matrix were 67.89% ± 9.14%, 78.12% ± 8.91%, 81.08% ± 8.99%, 89.12% ± 9.92%, and 92.96% ± 7.54%, respectively. According to the average classification results, the combination of the network attribute feature and the spatial feature of the adjacency matrix had the highest classification accuracies.

## 4. Discussion

Compared with the efficacy of traditional physical therapy, motor imagery therapy can induce brain plasticity, which can partially restore motor abilities of poststroke hemiplegic patients. At present, motor imagery therapy is mostly used for the recovery of upper-limb motor abilities but rarely for the recovery of lower-limb motor abilities. In addition, although there have been studies on the actual motor coordination of the lower limbs, few studies have investigated the neural mechanisms and decoding of imagined motor coordination of the lower limbs. Therefore, the focus of our present study was distinct from that of traditional studies on motor coordination, such that we focused on motor coordination imagery involving the lower limbs.

First, our present study was different from the simple motor imagery used traditionally for a unilateral limb, such as studies that focused on the upper limbs [58–64], as well as lower limbs [65–67]. Prior to our present study, no related studies on EEG-based lower-limb motor coordination have been published. In order to determine a rehabilitation method for lower-limb motor coordination in poststroke hemiplegic patients, we used a paradigm of motor coordination imagery involving the lower limbs that consisted of both normal gait imagery and poststroke hemiplegic gait imagery. Simple motor imagery of a unilateral limb mainly activates the contralateral brain area [68], while motor coordination imagery involving both lower limbs involves coordination of the left and right lower limbs, which requires the coordinated participation of relevant brain regions on the left and right sides of the brain. This kind of motor coordination imagery training may be beneficial for inducing plasticity of brain network structure/function or affected patients. In future research, we will design and improve our experimental research paradigm for poststroke hemiplegic patients with problems in motor coordination of the lower limbs.

The EEG pattern induced by motor coordination imagery involving the lower limbs is closely related to the performance of motor imagery psychological activities of subjects [68]. In order to ensure the effective completion of the motor coordination imagery involving the lower limbs and the reliability of the corresponding data, before the experiment, subjects were selected according to their scores on a motor-vision imagery questionnaire; subjects with scores higher than 70 were selected for participation in our present study. After the experiment, for the designed imagery task, each subject was required to fill in a questionnaire about the implementation of psychological activities and the degree of difficulty of imagination during completion of the experiments in our present study. The subjects reported that they could complete MCI and IEG in a controllable way, but that for MCI was easier to imagine compared to that for IEG. In future research, we intend to use online neurofeedback to further improve subjects' motor coordination imagery performance of the lower limbs.

In terms of analysis content, previous studies have mainly focused on the control of redundant degrees of freedom, quantification of motor coordination, external environments, and evaluation of motor coordination, while our present study focused on the neural mechanisms and decoding of motor coordination imagery involving the lower limb. In the EEG analysis method, in addition to traditional sensorimotor rhythm analysis [58–67], we also used MRCPs and brain networks to study motor coordination imagery involving the lower limbs. Previous studies have shown that MRCPs can represent neural activities related to planning before each exercise, the execution of each exercise, and the end of each exercise [64]. Previous studies have also shown that features of brain networks can represent the coordination among brain regions during activities such as sensation, perception, representation, and cognition [53]. The motor coordination imagery in our present study involved planning, execution, and termination of motor imagery, as well as coordination between brain regions.

In the time domain, the EEG patterns of IEG and REST were more consistent, while the EEG patterns of MCI and REST were almost opposite to one another, and the EEG patterns of MCI and IEG were also nearly opposite to one another. These findings may be due to the fact that the subjects were more familiar with resting states and walking states but were not as familiar with poststroke hemiplegic gaits. Unusual imagery can trigger different EEG patterns [36]. In our present study, the MRCPs of MCI and IEG were significantly different in terms of their EEG signals in the C3 and C4 channels. The EEG waveforms of MCI in the C3 and C4 channels were similar, but there was a significant difference at about 200 ms. The EEG waveforms of IEG in the C3 and C4 channels were nearly opposite to one another. The waveform of the C3 channel was a half cycle ahead of the waveform of the C4 channel, whereas they reached a consistent value at about 200 ms. This result may be due to MCI mainly involving bilateral lower limbs, while IEG mainly involves unilateral lower limbs. MRCPs will trigger ERP waveforms around 200 ms [35], so the ERP waveform is formed in the contralateral brain.

As demonstrated by brain topography, motor coordination imagery involving the lower limbs is mainly activated in sensorimotor areas, which is due to motor coordination imagery of the lower limbs representing a form of motor imagery, the latter of which has been shown to activate sensorimotor areas [10]. In addition to activating sensorimotor areas, the visual area located in the occipital lobe also has corresponding activation, which may be because the cues provided to the subjects are processed through the visual pathway before being transmitted to the sensorimotor area [68].

In the present study, power spectrum analysis showed that the power distribution related to MCI was distributed in *α* waves, while the power related to IEG was distributed in *θ* waves. Simultaneously, the absolute power of the IEG was much higher than the absolute power of the MCI. This result may be due to MCI being easier to complete than IEG, resulting in MCI inducing lower energy.

Under the condition of motor coordination or imagery, external stimulation acts on sensory organs and causes nerve excitation. The activation of brain-related areas and the coordinated control between brain areas transmit such nerve excitation to synergetic and antagonistic muscles, such that each muscle's activation is timed appropriately and ensures smooth completion of motor actions. Not only the activation of brain area but also the features of the brain network to represent the coordinated motion or imagination need to be calculated. In the present study, brain network analysis showed that brain functional connections during MCI, IEG, and REST mainly occurred in the right forehead, which may have been due to these psychological activities being related to the subconscious since human subconscious behavior is mainly related to the right forehead network [37, 53, 65, 69]. Our present brain network analysis also showed that the characteristic path length was a good distinguishing feature for MCI and IEG and that the other three measures were not. In addition, because the characteristic path length and the global efficiency determined the efficiency of the entire information transmission of the network [53], the characteristic path length and the global efficiency of the three types of tasks were quite different. IEG had the smallest characteristic path length and the highest global efficiency, so its network-information transfer rate was the fastest, followed by MCI; the slowest was REST, which may have been due to IEG needing to recruit more neural resources.

In the present study, the features of MRCPs, time-frequency analysis, power spectrum analysis, network attribute analysis, a combination of the network attribute features, and the spatial features of the adjacency matrix in MCI and IEG were classified by SVM, and the average classification accuracies were 67.89 ± 9.14%, 78.12 ± 8.91%, 81.08 ± 8.99%, 89.12 ± 9.92%, and 92.96 ± 7.54%, respectively. The results showed that the average classification accuracy of the combination of the network attribute features and the spatial features of the adjacency matrix was 92.96%, confirming that these were the two best classification features. This result may be due to features representing collaborative information among related brain regions; also, the feature dimensionality was high, so the relevant features were extracted and retained comprehensively.

Statistical analysis showed that there was a significant difference in the characteristic path lengths between MCI and REST, for which only the O2 channel was not statistically significant. There was no significant difference between the characteristic path lengths between IEG and REST, whereas the *p* values of P3, C3, CP4, and Pz channels of IEG were small, but the difference between MCI and IEG was significant. This result may be due to the fact that imagining MCI and REST is a relatively easy psychological activity, while imagining IEG is a relatively difficult psychological activity.

In Figure 5, there are some transition areas which are not clear by uncertainty. This is often more frequent in reality than one might think. We may segment each image obtained in order to delimit each activated area using a fuzzy procedure [70, 71].

## 5. Conclusion

In order to determine a rehabilitation method for lower-limb motor coordination in poststroke hemiplegic patients, a novel paradigm using lower-limb motor coordination imagery (normal gait imagery and poststroke hemiplegic gait imagery) was employed in the present study. Time-domain analysis, power spectrum analysis, time-frequency analysis, brain network analysis, and statistical analysis were used to investigate the neural mechanisms and decoding methods of motor coordination imaging of the lower limbs. The results showed that motor coordination imagery involving the lower limbs was mainly activated in sensorimotor areas and that the brain functional connection mainly occurred in the right forehead. The results also showed that the average classification accuracies of the combination feature of the network attributes and the spatial features of the adjacency matrix were 92.96% for normal gait imagery and poststroke hemiplegic gait imagery. Taken together, our findings may provide ideas for the rehabilitation of movement in poststroke hemiplegic patients based on motor coordination imagery involving the lower-limb brain-computer interface (BCI). Finally, our future directions are as follows: (1) EEG combined with fMRI to study the neural mechanisms of motor coordination imagery involving the lower limbs and (2) online verification of motor coordination imagery via BCI.

## Figures and Tables

**Table 1:** Results of repeated-measures one-way analysis of variance of characteristic path lengths of the 15 channels during MCI, IEG, and REST.

*p* values		REST														
		F3	F4	FC3	FC4	C3	Cz	C4	CP3	CP4	P3	Pz	P4	O1	O2	Oz
MCI	F3	0.000	0.000	0.001	0.000	0.000	0.002	0.001	0.000	0.000	0.000	0.000	0.000	0.000	0.000	0.000
	F4	0.000	0.002	0.007	0.000	0.002	0.014	0.009	0.000	0.000	0.001	0.000	0.001	0.002	0.000	0.000
	FC3	0.000	0.000	0.000	0.000	0.000	0.000	0.000	0.000	0.000	0.000	0.000	0.000	0.000	0.000	0.000
	FC4	0.000	0.000	0.000	0.000	0.000	0.000	0.000	0.000	0.000	0.000	0.000	0.000	0.000	0.000	0.000
	C3	0.000	0.000	0.000	0.000	0.000	0.001	0.000	0.000	0.000	0.000	0.000	0.000	0.000	0.000	0.000
	Cz	0.000	0.000	0.000	0.000	0.000	0.000	0.000	0.000	0.000	0.000	0.000	0.000	0.000	0.000	0.000
	C4	0.000	0.000	0.000	0.000	0.000	0.001	0.000	0.000	0.000	0.000	0.000	0.000	0.000	0.000	0.000
	CP3	0.000	0.000	0.001	0.000	0.000	0.002	0.001	0.000	0.000	0.000	0.000	0.000	0.000	0.000	0.000
	CP4	0.000	0.003	0.007	0.000	0.003	0.012	0.009	0.000	0.000	0.001	0.000	0.001	0.002	0.000	0.000
	P3	0.000	0.000	0.000	0.000	0.000	0.000	0.000	0.000	0.000	0.000	0.000	0.000	0.000	0.000	0.000
	Pz	0.000	0.000	0.000	0.000	0.000	0.000	0.000	0.000	0.000	0.000	0.000	0.000	0.000	0.000	0.000
	P4	0.000	0.000	0.000	0.000	0.000	0.000	0.000	0.000	0.000	0.000	0.000	0.000	0.000	0.000	0.000
	O1	0.000	0.000	0.000	0.000	0.000	0.000	0.000	0.000	0.000	0.000	0.000	0.000	0.000	0.000	0.000
	O2	0.031	0.160	0.277	0.000	0.174	0.359	0.312	0.063	0.044	0.113	0.045	0.154	0.199	0.011	0.022
	Oz	0.001	0.008	0.017	0.000	0.008	0.025	0.021	0.002	0.001	0.004	0.001	0.006	0.009	0.000	0.001
IEG	F3	0.000	0.005	0.012	0.000	0.005	0.019	0.016	0.001	0.000	0.002	0.000	0.002	0.005	0.000	0.000
	F4	0.002	0.039	0.104	0.000	0.041	0.162	0.127	0.005	0.003	0.017	0.003	0.023	0.048	0.001	0.003
	FC3	0.050	0.483	0.949	0.000	0.527	0.796	0.936	0.133	0.079	0.315	0.082	0.488	0.686	0.018	0.039
	FC4	0.034	0.226	0.422	0.000	0.244	0.549	0.478	0.074	0.048	0.148	0.051	0.213	0.299	0.014	0.025
	C3	0.000	0.003	0.009	0.000	0.003	0.017	0.012	0.000	0.000	0.001	0.000	0.001	0.003	0.000	0.000
	Cz	0.118	0.852	0.588	0.000	0.925	0.365	0.480	0.310	0.184	0.621	0.195	0.933	0.827	0.040	0.080
	C4	0.042	0.426	0.861	0.000	0.465	0.887	0.973	0.111	0.065	0.270	0.067	0.419	0.604	0.016	0.033
	CP3	0.021	0.214	0.454	0.000	0.232	0.622	0.531	0.048	0.031	0.121	0.033	0.181	0.291	0.009	0.017
	CP4	0.000	0.005	0.014	0.000	0.005	0.023	0.019	0.001	0.000	0.002	0.000	0.002	0.006	0.000	0.000
	P3	0.049	0.372	0.702	0.000	0.404	0.897	0.787	0.116	0.073	0.244	0.077	0.363	0.507	0.019	0.037
	Pz	0.000	0.006	0.017	0.000	0.007	0.029	0.023	0.001	0.000	0.002	0.000	0.003	0.007	0.000	0.000
	P4	0.019	0.257	0.595	0.000	0.285	0.833	0.699	0.046	0.030	0.134	0.031	0.213	0.363	0.008	0.017
	O1	0.074	0.644	0.821	0.000	0.703	0.568	0.701	0.198	0.117	0.440	0.122	0.679	0.911	0.026	0.054
	O2	0.028	0.156	0.273	0.000	0.166	0.348	0.309	0.059	0.040	0.108	0.042	0.148	0.199	0.011	0.019
	Oz	0.022	0.147	0.275	0.000	0.159	0.356	0.015	0.015	0.015	0.015	0.015	0.015	0.015	0.015	0.01

## Data Availability

The data used to support the findings of this study are available from the corresponding author upon request.

## References

[1] Teasell R. W., Bhogal S. K., Foley N. C., Speechley M. R. (2003). Gait retraining post stroke. *Topics in Stroke Rehabilitation*.

[2] Paolucci S., Bragoni M., Coiro P. (2008). Quantification of the probability of reaching mobility independence at discharge from a rehabilitation hospital in nonwalking early ischemic stroke patients: a multivariate study. *Cerebrovascular Diseases*.

[3] Flansbjer U. B., Holmbäck A. M., Downham D., Patten C., Lexell J. (2005). Reliability of gait performance tests in men and women with hemiparesis after stroke. *Journal of Rehabilitation Medicine*.

[4] Hutin E., Pradon D., Barbier F. (2012). Walking velocity and lower limb coordination in hemiparesis. *Gait & Posture*.

[5] Pollock A., Baer G., Pomeroy V., Langhorne P. (2007). Physiotherapy treatment approaches for the recovery of postural control and lower limb function following stroke. *Cochrane Database of Systematic Reviews*.

[6] Takeuchi N., Izumi S.-I. (2013). Rehabilitation with poststroke motor recovery: a review with a focus on neural plasticity. *Stroke Research and Treatment*.

[7] Belda-Lois J.-M., Mena-del Horno S., Bermejo-Bosch I. (2011). Rehabilitation of gait after stroke: a review towards a top-down approach. *Journal of NeuroEngineering and Rehabilitation*.

[8] Birbaumer N., Cohen L. G. (2007). Brain-computer interfaces: communication and restoration of movement in paralysis. *The Journal of Physiology*.

[9] Buch E., Weber C., Cohen L. G. (2008). Think to move: a neuromagnetic brain-computer interface (BCI) system for chronic stroke. *Stroke*.

[10] Luz Maria A. V., Ricardo Antonio S. R., Ramirez-Mendoza R. A. (2015). Motor imagery based brain-computer interfaces: an emerging technology to rehabilitate motor deficits. *Neuropsychologia*.

[11] Rossini P. M., Calautti C., Pauri F., Baron J.-C. (2003). Post-stroke plastic reorganisation in the adult brain. *The Lancet Neurology*.

[12] Caria A., Weber C., Brötz D. (2011). Chronic stroke recovery after combined BCI training and physiotherapy: a case report. *Psychophysiology*.

[13] Sitaram R., Veit R., Stevens B. (2012). Acquired control of ventral premotor cortex activity by feedback training. *Neurorehabilitation and Neural Repair*.

[14] Varkuti B., Guan C., Pan Y. (2012). Resting state changes in functional connectivity correlate with motor recovery for BCI and robot-assisted upper-limb training after stroke. *Neurorehabilitation and Neural Repair*.

[15] Wolpaw J. R., Birbaumer N., McFarland D. J., Pfurtscheller G., Vaughan T. M. (2002). Brain-computer interfaces for communication and control. *Clinical Neurophysiology*.

[16] Nilsen D. M., Gillen G., Gordon A. M. (2010). Use of mental practice to improve upper-limb recovery after stroke: a systematic review. *American Journal of Occupational Therapy*.

[17] Do A. H., Wang P. T., King C. E., Chun S. N., Nenadic Z. Brain-computer interface controlled robotic gait orthosis: a case report. http://arxiv.org/abs/1208.5024.

[18] Bernstein B. N. (1967). The co-ordination and regulation of movements. *Journal of Neuropathology & Experimental Neurology*.

[19] de Rugy A., Riek S., Oytam Y., Carroll T. J., Davoodi R., Carson R. G. (2008). Neuromuscular and biomechanical factors codetermine the solution to motor redundancy in rhythmic multijoint arm movement. *Experimental Brain Research*.

[20] Cheron G., Bengoetxea A., Dan B., Draye J. P. (1998). Multi-joint coordination strategies for straightening up motor in humans. *Neuroscience Letters*.

[21] Clark J. E., Phillips S. J. (1993). A longitudinal study of intralimb coordination in the first year of independent walking: a dynamical systems analysis. *Child Development*.

[22] Galloway J., Koshland G. (2002). General coordination of shoulder, elbow and wrist dynamics during multijoint arm movements. *Experimental Brain Research*.

[23] Kelso J. A. (1984). Phase transitions and critical behavior in human bimanual coordination. *American Journal of Physiology-Regulatory, Integrative and Comparative Physiology*.

[24] Shafir T., Brown S. H. (2009). Timing and the control of rhythmic upper-limb movements. *Journal of Motor Behavior*.

[25] Musselman K. E., Yang J. F. (2008). Interlimb coordination in rhythmic leg movements: spontaneous and training-induced manifestations in human infants. *Journal of Neurophysiology*.

[26] Van Emmerik R. (1990). Stability and instability in the coordination of multi joint limb motors. *Biology Neuroscience*.

[27] Diedrich F. J., Warren W. H. (1995). Why change gaits? dynamics of the walk-run transition. *Journal of Experimental Psychology: Human Perception and Performance*.

[28] Farmer S. E., Pearce G., Stewart C. (2008). Developing a technique to measure intra-limb coordination in gait: applicable to children with cerebral palsy. *Gait & Posture*.

[29] Sparrow W. A. (1992). Chapter 5 measuring changes in coordination and control. *Approaches to the Study of Motor Control and Learning*.

[30] Black D. P. (2005). Synergies in within- and between-person interlimb rhythmic coordination: effects of coordination stability and environmental anchoring.

[31] Kamm K., Thelen E., Jensen J. L. (1990). A dynamical systems approach to motor development. *Physical Therapy*.

[32] Lintern G. (1997). Dynamic patterns: the self‐organization of brain and behavior. *Complexity*.

[33] Miller R. H., Chang R., Baird J. L., Van Emmerik R. E. A., Hamill J. (2010). Variability in kinematic coupling assessed by vector coding and continuous relative phase. *Journal of Biomechanics*.

[34] Maffiuletti N. A., Bizzini M., Schatt S., Munzinger U. (2005). A multi-joint lower-limb tracking-trajectory test for the assessment of motor coordination. *Neuroscience Letters*.

[35] Jin J., Daly I., Zhang Y., Wang X., Cichocki A. (2014). An optimized ERP brain-computer interface based on facial expression changes. *Journal of Neural Engineering*.

[36] Scholz J. P., Schöner G. (1999). The uncontrolled manifold concept: identifying control variables for a functional task. *Experimental Brain Research*.

[37] Hackney M. E., Lim L. H., Jessica B., Bruce C., Mcgregor K. M. (2015). Context-dependent neural activation: internally and externally guided rhythmic lower limb motor in individuals with and without neurodegenerative disease. *Frontiers in Neurology*.

[38] Hua L., Yu-Qi C., Yang L. I., Tong Z., Xiang-Jiang R. (2017). Construct validity of Chinese version of kinesthetic and visual imagery questionnaire. *Chinese Journal of Rehabilitation Theory and Practice*.

[39] Alan D., Annette S., Dos A. S. M., Bastos C. A. (2018). A brazilian-Portuguese version of the kinesthetic and visual motor imagery questionnaire. *Arquivos de Neuro-Psiquiatria*.

[40] Chara P. J., Hamm D. A. (1989). An inquiry into the construct validity of the vividness of visual imagery questionnaire. *Perceptual and Motor Skills*.

[41] Marks D. F., David F. (1989). Bibliography of research utilizing the vividness of visual imagery questionnaire. *Perceptual and Motor Skills*.

[42] Marks D. F., David F. (1989). Construct validity of the vividness of visual imagery questionnaire. *Perceptual and Motor Skills*.

[43] Mckelvie S. J. (1990). The vividness of visual imagery questionnaire: commentary on the marks-chara debate. *Perceptual and Motor Skills*.

[44] Richardson J. T. E. (1995). Gender differences in the vividness of visual imagery questionnaire: a meta-analysis. *Journal of Mental Imagery*.

[45] Campos A., González M. A., Amor A. (2002). The Spanish version of the vividness of visual imagery questionnaire: factor structure and internal consistency reliability. *Psychological Reports*.

[46] Malouin F., Richards C. L., Jackson P. L., Lafleur M. F., Durand A., Doyon J. (2007). The kinesthetic and visual imagery questionnaire (kviq) for assessing motor imagery in persons with physical disabilities: a reliability and construct validity study. *Journal of Neurologic Physical Therapy*.

[47] Allbutt J., Shafiullah M., Ling J. (2006). The relationship between self-report imagery questionnaire scores and subtypes of socially desirable responding: visual and motor imagery. *Journal of Mental Imagery*.

[48] Campos A., Pérez-Fabello M. J. (2009). Psychometric quality of a revised version vividness of visual imagery questionnaire. *Perceptual and Motor Skills*.

[49] Randhawa B., Harris S., Boyd L. A. (2010). The kinesthetic and visual imagery questionnaire is a reliable tool for individuals with Parkinson disease. *Journal of Neurologic Physical Therapy*.

[50] Hideki N., Takayuki K., Kazumasa U., Satoru K., Shiori H., Shin M. (2018). Reliability and validity of the Japanese version of the kinesthetic and visual imagery questionnaire (kviq). *Brain Sciences*.

[51] Vidaurre C., Blankertz B. (2010). Towards a cure for BCI illiteracy. *Brain Topography*.

[52] Pfurtscheller G., Lopes da Silva F. H. (1999). Event-related EEG/MEG synchronization and desynchronization: basic principles. *Clinical Neurophysiology*.

[53] Li L., Caldwell G. E. (1999). Coefficient of cross correlation and the time domain correspondence. *Journal of Electromyography and Kinesiology*.

[54] Kay S. M. (1988). Modern spectral estimation. *Theory & Application*.

[55] Blinowska K. J. (2011). Review of the methods of determination of directed connectivity from multichannel data. *Medical & Biological Engineering & Computing*.

[56] Pichiorri F., Petti M., Morone G., Molinari M., Mattia D. (2014). Yia2: different brain network modulation following motor imagery bci-assisted training after stroke. *Clinical Neurophysiology*.

[57] Malik A. N., Iqbal J., Tiwana M. I. EEG signals classification and determination of optimal feature-classifier combination for predicting the movement intent of lower limb.

[58] Fong S., Ng S., Guo X., Wang Y., Ki W., Macfarlane D. (2016). Lower limb muscle reflex contraction latency, peak force and motor control in children with developmental coordination disorder. http://hdl.handle.net/10722/227730.

[59] Zhi X., Ming Z., Wong W. C., Zhang D., Sun Z., Jiang W. (2015). Inter-joint coordination of lower-limb kinematics between able-bodied person and transtibial amputee during walking. http://xueshu.baidu.com/usercenter/paper/show?paperid=1k710xx0p31q0xp0yk180gm069265067&site=xueshu_se&hitarticle=1.

[60] Stephenson J. L., Lamontagne A., Serres S. J. D. (2009). The coordination of upper and lower limb motors during gait in healthy and stroke individuals. *Gait and Posture*.

[61] Hutin E., Pradon D., Barbier F., Gracies J. M., Bussel B., Roche N. (2011). Lower limb coordination patterns in hemiparetic gait: factors of knee flexion impairment. *Clinical Biomechanics (Bristol, Avon)*.

[62] Silva M. G., Struber L., Brando J. G. T., Daniel O., Nougier V. (2018). Influence of dual-task constraints on the interaction between posture and movement during a lower limb pointing task. *Experimental Brain Research*.

[63] Priscila D. B. S., Oliveira A. S. C., Mrachacz-Kersting N., Kersting U. G. (2017). Effects of wobble board training on single-leg landing neuromechanics. *Scandinavian Journal of Medicine & Science in Sports*.

[64] Liepert J., Hassa T., Tüscher O., Schmidt R. (2011). Motor excitability during movement imagination and movement observation in psychogenic lower limb paresis. *Journal of Psychosomatic Research*.

[65] Rea M., Rana M., Lugato N. (2014). Lower limb movement preparation in chronic stroke. *Neurorehabilitation and Neural Repair*.

[66] Bose R., Khasnobish A., Bhaduri S., Tibarewala D. N. Performance analysis of left and right lower limb movement classification from EEG.

[67] Sueyoshi Y., Shimodozono M., Kawahira K., Yamashita M. (2017). Immediate effects of functional vibratory stimulation on the gait of stroke hemiplegia patients. *Journal of Neuroscience and Neuroengineering*.

[68] Turvey M. T. (1990). Coordination. *American Psychologist*.

[69] Debaere F., Assche D. V., Kiekens C., Verschueren S., Swinnen S. (2001). Coordination of upper and lower limb segments: deficits on the ipsilesional side after unilateral stroke. *Experimental Brain Research*.

[70] Versaci M., Morabito F. C. (2021). Image edge detection: a new approach based on fuzzy entropy and fuzzy divergence. *International Journal of Fuzzy Systems*.

[71] Versaci M., Morabito F. C., Angiulli G. (2017). Adaptive image contrast enhancement by computing distances into a 4-dimensional fuzzy unit hypercube. *IEEE Access*.

